# Th17 Cell Plasticity and Functions in Cancer Immunity

**DOI:** 10.1155/2015/314620

**Published:** 2015-10-25

**Authors:** Leslie Guéry, Stéphanie Hugues

**Affiliations:** Department of Pathology and Immunology, University of Geneva Medical School, 1211 Geneva, Switzerland

## Abstract

Th17 cells represent a particular subset of T helper lymphocytes characterized by high production of IL-17 and other inflammatory cytokines. Th17 cells participate in antimicrobial immunity at mucosal and epithelial barriers and particularly fight against extracellular bacteria and fungi. While a role for Th17 cells in promoting inflammation and autoimmune disorders has been extensively and elegantly demonstrated, it is still controversial whether and how Th17 cells influence tumor immunity. Although Th17 cells specifically accumulate in many different types of tumors compared to healthy tissues, the outcome might however differ from a tumor type to another. Th17 cells were consequently associated with both good and bad prognoses. The high plasticity of those cells toward cells exhibiting either anti-inflammatory or in contrast pathogenic functions might contribute to Th17 versatile functions in the tumor context. On one hand, Th17 cells promote tumor growth by inducing angiogenesis (via IL-17) and by exerting themselves immunosuppressive functions. On the other hand, Th17 cells drive antitumor immune responses by recruiting immune cells into tumors, activating effector CD8^+^ T cells, or even directly by converting toward Th1 phenotype and producing IFN-*γ*. In this review, we are discussing the impact of the tumor microenvironment on Th17 cell plasticity and function and its implications in cancer immunity.

## 1. Introduction

CD4^+^ T helper (Th) cells represent an essential component of adaptive immunity since they are absolutely necessary to regulate CD8^+^ T cells and B cells responses and to induce late recruitment of innate immune cells at inflammatory sites. Although originally defined as Th1 and Th2 subsets, new Th CD4^+^ T cell subsets emerged the last decades such as suppressive Treg cells and proinflammatory Th17, and more recently for Th9, Th22, TR1, and TFH cells. Although Th1 and Th2 subsets are considered as definitive and mutually exclusive lineages, it seems that Th17 and Treg subsets do not represent stable differentiation processes and retain plasticity allowing them to adapt to different environments.

Th17 cells were first characterized in 2005 as a Th cell lineage independent from Th1 and Th2 subsets [1, 2]. Th17 cells are defined by their production of IL-17 (also known as IL-17A), although they also produce IL-17F, IL-21, GM-CSF, and IL-22 [3]. Engagement of naïve CD4^+^ T cells into the Th17 subset depends on different cytokine cocktails including TGF-*β*, IL-6, IL-1*β*, or IL-21 [3]. Although not required for Th17 cells differentiation, IL-23 was shown to maintain their pathogenic phenotype and survival [4]. Ror*γ*t [5], or its homolog Rorc in human [6], is the most specific transcription factor promoting Th17 cell differentiation, although it also relies on additional transcription factors such as Ror*α* [7], Stat3 [8], BATF [9], IRF4 [10], and AhR [11, 12]. Upon steady state, Th17 cells are located in lamina propria of the small intestine but can be induced in any other tissues (more precisely in mucosal and epithelial barriers) to fight extracellular bacteria, viruses, and fungi [13]. Indeed, IL-17 induces inflammatory cytokines (namely, TNF, IL-1*β*, and IL-6), colony-stimulating factors (G-CSF and GM-CSF), and chemokines (CXCL-8 and CXCL-2) production, leading to granulopoiesis and granulocyte recruitment at inflamed sites [14–16]. Moreover, and together with IL-22, IL-17 induces antimicrobial peptides and proteins (*β*-defensins and S100 proteins) production by keratinocytes [17]. Importantly, Th17 cells were shown to act as bona fide Th cells by enhancing B cell [18] and CD8^+^ T cell [19, 20] responses. However, Th17 cells are associated with inflammatory and autoimmune diseases in mice and human. Notably, antigen-specific Th17 cells and their related cytokines are highly pathogenic and exhibit detrimental roles in multiple sclerosis, psoriasis, systemic lupus erythematosus, rheumatoid arthritis, inflammatory bowel disease, and asthma [3]. While Th17 cells function as pathogenic Th cells in autoimmunity, their role in cancer is still under debate. In addition, whether Th17 plasticity and conversion into several Th cells, will, as described in many inflammatory diseases, similarly happen in tumor context will be discussed in this review.

## 2. Th17 Cell Plasticity

**Figure 1:**
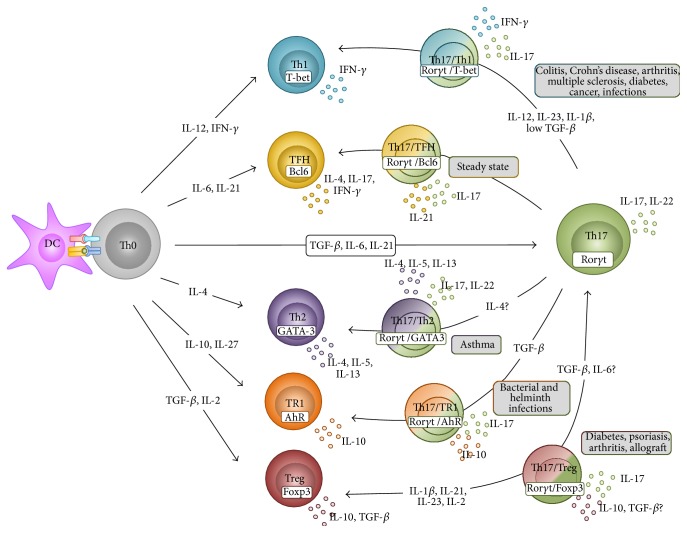
Th17 cell plasticity. T helper cells differentiate from naïve T cells. Th17 cells are endowed with the capacity to convert toward different other lineage subsets, depending on the microenvironment. Upon steady state Th17 cells constantly convert toward TFH and participate in the development of IgA-secreting germinal center B cells. In addition, Th17 cells acquire pathogenic functions by converting toward Th1 cells during autoimmunity, cancer, and infections or toward Th2 cells during asthma. Alternatively, Th17 cells gain immunosuppressive functions by converting toward Foxp3^+^ Treg cells or TR1 cells in the context of autoimmune diseases or infections.

In contrast to Th1 and Th2 cells that are considered as stable lineages, Th17 cells exhibit high degree of plasticity. Th17 cells can mainly transdifferentiate into Th1 or Treg cells, but also into TR1, Th2, or TFH cells endowing them with multiple and opposing functions, and consequently allowing them to elicit qualitatively distinct responses depending on different microenvironments. Th17 plasticity is summarized in Figure 1.

### 2.1. Th17/Th1 Cell Plasticity

In human, hybrid cells producing both IFN-*γ* and IL-17 and coexpressing Th17 and Th1-related transcription factors (namely, Ror*γ*t or Rorc and T-bet, resp.) were described in many inflammatory autoimmune diseases such as Crohn's disease [6], rheumatoid arthritis [21], and multiple sclerosis [22].* In vitro* experiments suggested that in the presence of low amounts, or in total absence of TGF-*β*, IL-12 and IL-23 cytokines induced the conversion of Th17 cells toward a Th1 phenotype whereas sufficient TGF-*β* quantities maintained a Th17 phenotype [6, 21, 23]. In addition, Smad7 (an intracellular TGF-*β* inhibitor) overexpression in Th17 cells resulted in an enhanced conversion toward Th1 cells, suggesting that TGF-*β* inhibits such plasticity [24]. Treatment of* in vitro* polarized Th17 cells with a combination of IL-12 and IL-23 abrogated IL-17 production and in contrast enhanced IFN-*γ* secretion by Th17 cells, in a mechanism dependent on the Th1-related transcription factors Stat-4 and T-bet [23]. In agreement, Th17/Th1 hybrid cells were found in elevated levels in the synovial fluid compared to the blood of juvenile idiopathic arthritis patients and were associated with increased IL-12 and decreased TGF-*β* levels (IL-23 was not detectable) [21]. The conversion of Th17 cells exposed to arthritic synovial fluid into Th1 cells was blocked when IL-12 was inhibited in the culture [25] suggesting that the joint microenvironment was responsible for Th17/Th1 cell plasticity through a mechanism involving IL-12 [21, 25]. Similarly, Th17/Th1 hybrid cells were easily detectable in the gut of Crohn's disease patients. Furthermore, Th17 clones derived from Crohn's disease patients' gut exhibited Th1 cell conversion when treated with IL-12* in vitro*, as demonstrated by a decrease in Ror*γ*t expression and IL-17 production and an increase in IFN-*γ* production [6].

In mice,* in vitro* polarized Th17 cells transferred in Rag^−/−^ mice converted into Th1-like cells, characterized by IFN-*γ* production, and resulted in colitis [23]. Similarly,* in vitro* Th17 polarized BDC2.5 TCR transgenic CD4^+^ T cells (expressing a TCR specific for a pancreatic *β*-cell antigen, the chromogranine A) transferred in NOD-SCID recipients exhibited conversion into Th1 cells and consequently induced type 1 diabetes [26]. In addition, using IL-17^+^ cell fate mapping reporter mice, Hirota et al. demonstrated that IFN-*γ* producing CD4^+^ T cells in spinal cords of experimental autoimmune encephalomyelitis (EAE) mice (a mouse model for multiple sclerosis) almost all derived from ex-Th17 cells, although they have stopped producing IL-17 [27]. Conversion was shown to rely on IL-23 since the IL-23 deficient mice, although displaying similar levels of Th17 cells, lacked Th17/Th1 subsets and “ex-Th17” Th1 cells. The absence of IL-23 appeared to prevent T-bet upregulation and consequently to inhibit Th17 cell conversion toward a Th1 phenotype. However, overexpression of T-bet in Th17 cells was clearly not sufficient to drive Th1 conversion, suggesting that additional partners might be required [28]. Accordingly, it has been recently shown that the generation of Th17/Th1 hybrid cells required not only T-bet but also Runx1 or Runx3 [28]. Runx1 bound to* Ifng* locus in a T-bet-dependent manner in IL-12-stimulated Th17 cells and induced Th17 toward Th1 plasticity [28]. Altogether, those studies demonstrate that IL-12 and/or IL-23 are likely to be responsible for Th17 cell conversion toward Th1 cells during autoimmune disease progression.

In human, some* Candida albicans*-specific Th17 cells produced both IL-17 and IFN-*γ*, but not IL-10, whereas* Staphylococcus* aureus-specific Th17 cells produced IL-17 and IL-10 upon restimulation [29], thus demonstrating that plasticity can allow Th17 cells to promote different responses toward various pathogens. Moreover, upon* Candida albicans* infection, IL-1*β* was shown to be essential to drive IFN-*γ* production by Th17 clones whereas, in the same experimental settings, and in contrast to what was shown using autoimmune mouse models, IL-12 was inhibiting Th17/Th1 conversion [29]. Those results demonstrate that, although Th17/Th1 cells are readily detected in different microenvironments established under autoimmune or inflammatory conditions, the mechanisms accounting for their generation might differ from one condition to another.

While Th17 cells seem to easily convert toward a Th1 phenotype, Th1 cells are considered stable and mostly refractory to conversion toward Th17 cells or other Th subsets, suggesting that plasticity between Th1 and Th17 cells is rather asymmetric. In agreement, the study of epigenetic marks in various Th cell subsets revealed that while Th1 cells exhibit a permissive status on Th1 genes and silencing marks on other lineage genes, Th17 cells might retain bivalent status on Th1 genes such as Tbx21 (encoding for the transcription factor T-bet), allowing further plasticity toward Th1 cell subset [30]. New pieces of data recently challenged this dogma. Microbiota-Ag specific Th1 cells adoptively transferred into Rag^−/−^ mice converted into Th17 cells and drove colitis [31]. In this study, however, Th1 cells converted into Th17 cells in absence of the endogenous T cell compartment, and those findings need therefore to be confirmed in physiological conditions before concluding any Th1 plasticity toward Th17 phenotype.

### 2.2. Th17/Treg Cell Plasticity

Th17 and Treg CD4^+^ T cells subsets partially share differentiation programs. Indeed, TGF-*β* alone drives Treg cell differentiation while it induces Th17 cell differentiation and inhibits Treg cell differentiation in the presence of other cytokines such as IL-6 or IL-21 [3]. Various factors were shown to regulate the fate of CD4^+^ T cells towards Th17 or Treg subsets, including not only retinoic acid [32] or AHR [11, 12], but also glucose metabolism via HIF1a [33, 34] or fatty acids metabolism [35, 36]. Interestingly,* Lactobacillus reuteri* given in drinking water induced an increase in Treg cells and a decrease in Th17 cells and resulted in reduced obesity in mice [37], demonstrating a control of Treg/Th17 balance in gut immunity by probiotics. Due to this close relationship between Treg and Th17 cells, plasticity between these two subsets was easily observed and extensively described in mice and in humans. Many studies reported the production of IL-17 by Treg cells, associated with a decrease in Foxp3 and a concomitant increase in Ror*γ*t (or Rorc in human) expressions [38–40], thus demonstrating a switch toward Th17 cell subset* ex vivo* and* in vivo*. However, depending on the studies, those hybrid cells (Foxp3^+^ Ror*γ*t^+^ CD4^+^ T cells) could either retain or lose immunosuppressive capacities, possibly depending on Foxp3 expression levels [39, 41]. Moreover, “ex-Foxp3” cells differentiated toward a Th17 phenotype might play an important role in autoimmunity, as demonstrated in type 1 diabetes mouse model [42]. Treg cells extracted from psoriatic patient blood revealed higher susceptibility to convert toward Th17 cells than Treg cells from the blood of healthy donors, and Foxp3^+^ IL-17^+^ CD4^+^ cells were detected in psoriatic lesions [43]. In a mouse model of rheumatoid arthritis, Foxp3 fate reporter mice revealed that “exFoxp3^+^” cells converted toward Th17 cells under IL-6 exposure in the synovia and became highly osteoclastogenic [44]. IL17^+^ Foxp3^+^ T cells were also detected in the synovia of patients with active rheumatoid arthritis [44]. On the opposite side, conversion of Th17 cells toward a Treg phenotype has also been described, demonstrating that plasticity between Treg and Th17 cells is a two-way process. In IL-17 fate reporter mice, when allograft survival was induced by the transfer of mesenchymal stem cell in combination with immunosuppressive drugs, Th17 cells could give rise to either double IL17^+^Foxp3^+^ cells or IL-17^−^Foxp3^+^ cells, thus confirming the conversion of Th17 cells toward a Treg phenotype [45]. Therefore, factors influencing Treg versus Th17 differentiation, or Treg/Th17 plasticity, might represent interesting targets to manipulate immune responses toward immunogenicity in cancer or in contrast toward tolerance in autoimmune diseases.

### 2.3. Th17/TR1 Cell Plasticity

In a model of tolerance induced by the injection of an anti-CD3 antibody, Th17 cells recruited in the small intestine acquired immunosuppressive functions dependent on IL-10, TGF-*β*, and CTLA-4 [46]. This study suggested that Th17 cells in the small intestine exhibit some features of TR1 cells. Accordingly, using fate reporter mice, the same team has further recently shown that Th17 cells could convert toward a TR1 phenotype. Indeed, both upon steady state and after immune response induction (including anti-CD3 mAbs treated EAE mice,* N. brasiliensis* helminth infection and* S. aureus* bacterial infection), some ex-Th17 cells produced IL-10 (without expressing Foxp3), expressed the TR1 markers LAG-3, exhibited a gene expression profile similar to TR1 cells, and acquired immunosuppressive functions. In agreement, TGF-*β* and downstream Smad3 and AhR were shown to support the conversion of Th17 to TR1 cells [47].

### 2.4. Th17/Th2 Cell Plasticity

In addition to Th17/Th1 and Th17/Treg hybrids cells, Th17/Th2 cells were described in blood of asthma patients. Those cells exhibit features of both Th17 and Th2 lineages, that is, the expression of transcription factors GATA3 and Ror*γ*t and the secretion of the cytokines IL-17, IL-22, IL-4, IL-5, and IL-13 [48, 49]. Using a mouse model for lung allergic disease, those cells were reported to be more pathogenic by inducing profound influx of inflammatory leukocytes and consequently leading to asthma exacerbation [48]. Moreover, it was demonstrated* in vitro* that Th17 cells can acquire Th2 features whereas the opposite could not occur [50] and that IL-4 could be responsible for Th17 plasticity toward Th2 phenotype [49].

### 2.5. Th17/TFH Cell Plasticity

Recently, it was demonstrated that Th17 and TFH cells, at least in human, shared common early differentiation paths [51]. Moreover, Th17 cells were shown to convert toward TFH phenotype in Peyer's patches. Indeed, using IL-17 fate reporter mice, it was demonstrated that, in steady state, Th17 cells continuously acquire a TFH phenotype (expression of Bcl6, CXCR5, PD1, and IL-21) in Peyer's patches and induce the development of IgA-secreting germinal center B cells [52].

## 3. Th17 Cells in Cancer

Th17 cells are often associated with tumors. Indeed, tumor-infiltrating Th17 cells were reported for many cancers in mice and humans, including melanoma, breast, colon, hepatocellular, ovarian, pancreatic, prostate, and renal tumors [53]. Moreover, Th17 cells accumulate specifically in many different tumors (esophageal carcinomas, breast, colon cancers, and melanoma) compared to healthy tissues [54–57], demonstrating a specific recruitment of Th17 cells by the tumor microenvironment itself. However, it is still unknown whether Th17 cells are induced, recruited, expanded, or converted from Tregs in tumors. It is likely that all of these processes coexist. Intratumoral recruitment of Th17 cells was proposed to rely on various chemokines depending on the tumor context, such as CCL20 [58], CCL17, CCL22 [56], MIF [57], RANTES, MCP1 [55], or CCL4 produced by immature myeloid cells [59]. Moreover, cancer cells, tumor-derived fibroblasts, and antigen-presenting cells secrete several key cytokines for Th17 differentiation such as IL-1*β*, IL-6, IL-23, and TGF-*β*. In the tumor, IL-1*β*, probably produced by tumor-associated macrophages, was shown to be critical for the expansion of memory Th17 cells in ovarian and breast cancers [54, 60]. In mammary gland tumors, PGE2-induced IL-23 production led to Th17 cell expansion [61]. In addition, in particular experimental conditions in mice (IDO inhibition combined with vaccination protocols), Th17 cells could arise from Treg conversion although we ignore if this could happen in a basal tumor microenvironment [62].

Intratumoral Th17 cell infiltration has been associated with both good and bad prognoses. Indeed, Th17 cell infiltration in human tumors was correlated with better survival in ovarian cancer patients [54], prostate cancer patients [63], lung carcinoma, and squamous cell carcinoma patients [64] or with bad prognosis in hepatocellular [65], colorectal [66], pancreatic [67], and hormone resistant prostate carcinoma patients [68]. Some reviews nicely summarized the different correlations between Th17 cells infiltration and prognosis in human cancers [69, 70]. Contradictory results also emerged from mice deficient for IL-17 or IL-17R. Indeed, some studies reported increased tumor growth in absence of IL-17 in B16 melanoma and MC38 colon carcinoma models [19, 71]. On the opposite side, IL-17 deficiency led to decreased tumor growth in B16 melanoma and MB49 bladder carcinoma models [72] and IL-17R^−/−^ mice exhibited decreased tumor growth, when challenged with EL4 lymphoma, Tramp-C2 prostate cancer, or B16 melanoma tumor cells [73]. Similarly, IL-17 overexpressing tumors exhibited either enhanced [74, 75] or decreased tumor growth in mice [76].

## 4. Th17 Cell Derived Cytokines and Angiogenesis

IL-17, the Th17 hallmark, was often correlated with high vascular density and VEGF production within tumors, suggesting that IL-17 promotes angiogenesis. Indeed, in mice, IL-17 overexpressing tumors grew more and exhibited higher vascular density [76, 77]. It was demonstrated that IL-17 induces production of VEGF and other angiogenic factors by tumors cells and fibroblasts [76]. In addition, in B16 melanoma and MB49 bladder carcinoma models, IL-17 induced IL-6 production by tumor cells which, in turn, activated Stat3-dependent survival and angiogenic genes expression [72]. In human, IL-17 and angiogenesis were correlated in gastric [78], colorectal [79], hepatocellular [65], breast [80], lung [81], and pancreatic tumors [67]. However, in ovarian cancer, IL-17 production was associated with antiangiogenic chemokines and reduced tumor growth [71]. Moreover, in mouse models, IL-17 promoted MDSC recruitment within tumors [81] or development and suppressive MDSC functions [73], indicating additional protumoral roles for IL-17. Besides IL-17, Th17 cells produce other cytokines, including IL-17F and IL-21, that have been shown to exhibit antiangiogenesis functions and to play protective roles against tumor development [82, 83]. In those studies, however, IL-17 production was not always correlated to Th17 cells since CD68^+^ macrophages [80], neutrophils [84], MDSCs [85], *γδ* T cells [81], endothelial cells, stromal cells, and tumors cells [53] can produce IL-17. A recent study has determined that Th17 represent only a minor fraction of IL-17 expressing cells in different human tumors and that IL-17 was mainly produced by neutrophils or mast cells [84]. Moreover, in squamous cervical cancer, IL-17 was correlated with poor prognosis whereas Th17 cell infiltration was associated with better outcome [84]. A systematic review of the literature established that IL-17 was indeed related to bad prognoses but Th17 cells frequencies were correlated with improved prognosis in tumors in general [69]. However, although it is clear that a distinction has to be made between IL-17 and Th17, some discrepancies remain and the impact of Th17 cells might differ depending on the inflammatory context and tumor type.

## 5. Th17 Cell Immunosuppressive Functions in Tumor Context

### 5.1. Th17 Cell Plasticity

Alternative immunosuppressive mechanisms might account for protumoral functions of Th17 cells. It is quite puzzling that, in contrast to other inflammatory situations, evidence for acquisition of immunosuppressive functions by Th17 cells converting towards Treg lineage in tumor context is rather limited. Indeed, human TILs-derived Th17 clones, characterized by IL-17 production and Ror*γ*t expression and cultured* in vitro* to maintain their phenotype (on OKT3 cells and allogeneic PBMCs), naturally converted into Treg cells upon TCR engagement, acquiring both Foxp3 expression and* in vitro* immunosuppressive functions. Importantly, this transdifferentiation appeared to be very stable since Th17-derived Treg cells were refractory to return conversion toward Th17 phenotype in presence of Th17 polarizing cytokines [86]. However, whether Th17 cells actually convert toward Treg phenotype* in vivo* in a tumor microenvironment is still unknown. In addition, although Th17/Treg (IL-17^+^Foxp3^+^) hybrid cells have been described in human tumors, they mostly originate from bona fide Treg cells [87]. Those immunosuppressive IL17^+^ Foxp3^+^ T cells were described for instance in human colorectal and esophageal cancers, but not in ovarian cancer, melanoma, or renal cell carcinoma [87–91]. When extracted from colorectal cancer biopsies, IL17^+^ Foxp3^+^ T cells promoted tumorigenicity in spheres forming stem cells [90] and inhibited tumor-specific CD8^+^ T effectors [89]. In contrast, in a melanoma mouse model, Treg cells converted into Th17 cells exhibiting antitumoral effects. Indeed, CpG-activated plasmacytoid dendritic cells (pDCs) expressing IDO prevented Treg conversion. However, when IDO was inhibited in pDCs, they produced IL-6 and consequently promoted Treg plasticity toward Th17 cells [62, 92]. In a mouse model of established melanoma, this conversion was associated with enhanced CD8^+^ T cells activation and reduced tumor growth [62]. Thus, studies describing Th17 plasticity in the tumor context are rather sparse and require further confirmation before determining whether they originate from Treg or Th17 cells, and more importantly, before claiming an important role for those cells in tumor immunity.

### 5.2. Other Th17 Cell Immunosuppressive Functions

In addition to potential cell plasticity, Th17 cells may also exert their immunosuppressive functions via ectonucleotidases CD39 and CD73. CD39 converts ADP or ATP into AMP, and CD73 converts AMP into adenosine that exhibits immunosuppressive functions by inhibiting T cell proliferation and cytokine production [93] and therefore represents a major mechanism for Treg-mediated immunosuppression [94].* In vitro*, TGF-*β*+IL-6 polarized Th17 cells express the ectonucleotidases CD39 and CD73, while it is not the case when Th17 cells are polarized with the cytokines IL-6, IL-23, and IL-1*β* [95]. CD39 and CD73 conferred immunosuppressive functions to Th17 cells toward Tc1 and Th1 cells* in vitro*.* In vivo*, the transfer of CD39^+^ CD73^+^ Th17 cells, polarized* in vitro* using TGF-*β*+IL-6, promoted tumor growth. Interestingly, those cells were Foxp3 negative and do not represent a conversion of Th17 toward Treg phenotype [95]. Altogether, these data determined that Th17 cells can support tumor growth by promoting angiogenesis and/or inhibiting immune responses via Treg conversion or ectonucleotidases expression.

## 6. Th17 Cell Antitumor Functions

### 6.1. Th17 Cells Roles in Recruitment and Activation of Effector Cells in Tumors

In addition to protumoral roles described for IL-17 and Th17 cells, many reports have demonstrated that Th17 cells also drive antitumoral immunity. First of all, tumor growth was increased in both IL-17^−/−^ (B16 melanoma and MC38 colon cancer cell lines) [19, 71] and Ror*γ*t^−/−^ mice (B16 melanoma cell line) [96]. In IL-17^−/−^ mice, enhanced tumor growth and lung metastases were associated with decreased IFN-*γ* ^+^ NK cells and IFN-*γ* ^+^ T cells in tumor draining lymph nodes and in the tumor itself [71], strongly suggesting a protective role for endogenous Th17 cells.

Moreover, transfer of* in vitro* polarized Th17 cells induced established tumor regression or reduced number of tumor foci in B16 melanoma model [19, 20, 97, 98]. Although Th17 cells do not exhibit direct killing activity [20], several mechanisms for antitumor Th17-mediated effects were proposed. It was shown that Th17 cells induced recruitment and activation of CD8^+^ T cells in the tumor [19]. Tumor infiltrating Th17 cells induced CCL20 production, thus promoting DC recruitment within the tumor and subsequent migration to draining lymph nodes of tumor material containing DCs, leading to potential activation of CD8^+^ T cells [19]. Another study showed that Th17 cells might directly and indirectly activate CD8^+^ T cells in tumor context. After* in vitro* coculture in presence of DC expressing tumor antigens, activated Th17 cells indeed acquired MHCI-peptide complexes from DCs and directly activate CD8^+^ T cells through MHCI-TCR interaction and IL-2 production. In addition, the same study showed that transferred Th17 cells promoted the recruitment of immune cells within the tumors, including CD4^+^ T cells, CD8^+^ T cells, and DCs, potentially through the Th17-induced chemoattractants CCL20 and CCL2 [20]. In addition, we have recently demonstrated that upon immunization, tumor Ag presenting pDCs induced Th17 cells that promote massive and general intratumor immune cell recruitment, including CTLs, and resulted in tumor rejection [99]. This further confirmed the implication of Th17 cells in immune effector cells recruitment within tumors after either T cell transfer [19, 20] or vaccination [99].

### 6.2. Th17 Cell Plasticity in Tumors

As described in many contexts, Th17/Th1 cells were also associated with tumors. Kryczek et al. analyzed Th17 cells in human ovarian tumors. IL-17 was almost exclusively produced by CD4^+^ T cells, and those Th17 cells also expressed CXCR4, CCR6, and CD161. In addition, all IL-17 producing Th17 cells also produced IL-2 and TNF, and for a significant fraction, IFN-*γ* [54]. In line with a role for Th17 cells in the recruitment of immune cells within tumors, Th17 cells in human ovarian cancers were positively correlated with IFN-*γ* ^+^ CD4^+^ T cells, IFN-*γ* ^+^ IL-17^+^ CD4^+^ T cells, and IFN-*γ* ^+^ CD8^+^ T cells, whereas negatively correlating with Treg cells. IL-17 and IFN-*γ* synergistically induced CXCL9 and CXCL10 production by tumor cells, possibly leading to increased CD8^+^ T cell infiltration within tumors [54]. Importantly, another study has identified tumor antigen-specific Th17/Th1 cells in human lung tumors [100]. Adoptive transfer of* in vitro* polarized tumor antigen-specific (tyrosinase-related protein 1, TRP-1) Th17 cells into B16 melanoma tumor bearing mice demonstrated that Th17 cells were more potent to induce tumor rejection compared to Th1 cells. Moreover, Th17 antitumoral effect was strictly dependent on their capacity to produce both IFN-*γ* and IL-17. Indeed, transfer of IL-17A^−/−^ Th17 cells, IFN-*γ* ^−/−^ Th17 cells, and Tbx21^−/−^ Th17 cells into WT mice or transfer of WT Th17 cells into IFN-*γ*-R^−/−^ recipient mice failed to control tumor growth [97, 98]. IFN-*γ* exhibits many antitumoral activities, either by directly exerting antiproliferative, proapoptotic, and antiangiogenic functions, or by indirectly activating cytotoxic functions of monocytes/macrophages, NK cells, or CD8^+^ T cells [101, 102]. Moreover, adoptive transfer of CD4^+^ T cells overexpressing Smad7, an intracellular inhibitor of TGF-*β* signaling, resulted in increased number of tumor-infiltrating Th17/Th1 hybrid cells and inhibition of tumor growth. Those cells were characterized by expression of both T-bet and Ror*γ*t, decreased IL-17, increased IFN-*γ*, and TNF-*α* production. Smad7 overexpressing T cells further exhibited direct killing of tumor cells via TNF-*α*, thus demonstrating an additional mechanism accounting for Th17/Th1 hybrid cell antitumor functions [24]. In addition, Th17 cells maintain a molecular transcriptional profile distinct from Th1 cell derived counterparts but exhibit stem cell-like signature. Th17 cells are consequently endowed with enhanced capacities to survive and self-renew, generate effector progeny, and enter the memory pool with efficiency superior to that of Th1 cells [98]. Those characteristics might explain why Th17 cells can be so efficient at rejecting tumors in transfer models.

**Figure 2:**
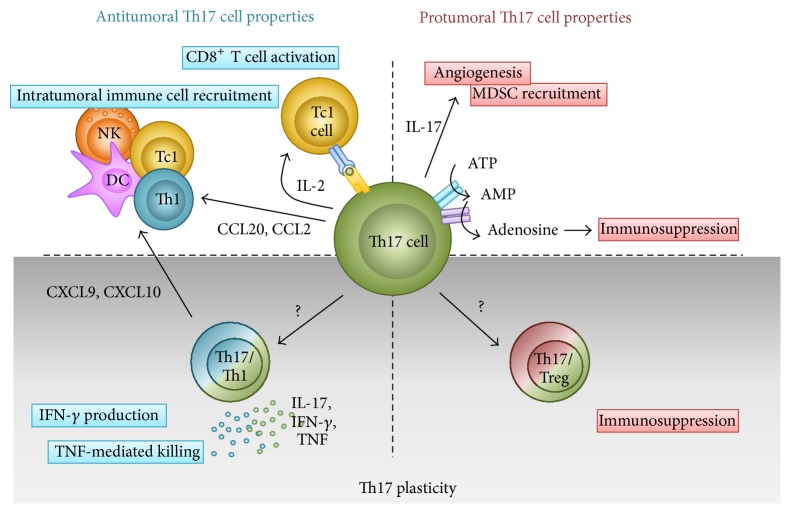
Roles of Th17 cells in tumor immunity. Depending on their plasticity (upper panels), Th17 cells exhibit both pro- and antitumoral functions. IL-17 production by Th17 cells might contribute to angiogenesis and intratumoral MDSC recruitment. Moreover TGF-*β* might induce Immunosuppression in Th17 cells by inducing ectonucleotidases expression. On the contrary, Th17 cells were shown to inhibit tumor growth by inducing immune effector cell recruitment within tumors and also by activating tumor-specific cytotoxic CD8^+^ T cells. Plasticity (lower panels) might confer additional functions to Th17 in tumor immunity. Whether Th17 cells can actually convert toward Treg cell phenotype in the tumor microenvironment requires further confirmation but might confer immunosuppressive functions to Th17 cells. On the opposite side, Th17 cells convert toward a Th1 cell phenotype and produce IFN-*γ* and TNF-*α* in the tumor that will result in tumor growth inhibition.

How the tumor microenvironment will impact T cell plasticity remains to be investigated. Whether Th17 cells will convert toward Th1 cells locally within the tumor or whether Th17/Th1 hybrid cells will be recruited within the tumor is unknown. As mentioned above, studies have identified IL-12, IL-23, IL-1*β*, and TGF-*β* as regulators of Th17/Th1 cell conversion in several immunological contexts but not in cancer. The production of IL-1*β* or IL-23 by macrophages in the tumors might play a role* in situ*. However, IL-12 amounts are usually low in tumors, which might not favor Th17/Th1 cell conversion. In addition, TGF-*β* known to inhibit such a conversion is often highly expressed in tumors [103, 104]. Altogether, these studies have identified different mechanisms by which Th17 cells are controlling tumor growth as follows: recruitment of several immune cells including DCs, CD4^+^ T cells, and CD8^+^ T cells within tumors, activation of CD8^+^ T cells, and possibly plasticity toward Th1 phenotype, associated with IFN-*γ* and TNF-*α* production. Pro- and antitumoral functions of Th17 cells are summarized in Figure 2.

## 7. Concluding Remarks

As discussed herein, Th17 cell functions in tumor immunity are still ambiguous and remain difficult to appraise. Future work aiming at understanding how Th17 cells are regulated in tumor context should determine how and where Th17 cells are primed and function. Both the tumor type and the progression stage are highly influencing the tumor microenvironment and thereby will subsequently impact Th17 cell plasticity. Th17 cells will acquire either immune suppressive functions or antitumoral capacities, leading to tolerance toward tumors or antitumoral immune responses, respectively.

Th17/Th1 plasticity represents an attractive target for cancer immunotherapies. Indeed, manipulations aiming at enhancing this conversion, or constraining its inhibitors, might result in a better tumor growth control. IL-12 has been extensively studied, since it might provide antitumor effects by enhancing IFN-*γ* production. Clinical studies have however been disappointing since systemic treatments with recombinant IL-12 exhibited cytotoxicity and gave rise to small beneficial impacts. Recent clinical trials are currently taking advantage of IL-12 antitumoral effects while trying to limit its cytotoxicity by delivering the cytokine directly at the tumor site [105]. Alternatively, although endogenous IL-23 was shown to display protumoral effects, exogenous IL-23 has demonstrated antitumoral functions and might represent as well an interesting immunotherapeutic axis [106]. Finally, blocking TGF-*β* might allow Th17 conversion toward Th1 while inhibiting immunosuppressive Th17 cell functions. Regarding TGF-*β* implication in promoting metastases [107], blocking this cytokine could improve cancer therapies in two ways, by directly inhibiting distal tumor propagation and by improving antitumor immunity.

In addition, Th17 cell transfer has shown incredible efficiency to treat established tumors in mouse models, and translation into humans therefore represents promising although challenging future cellular therapies.* In vitro*, polarized Th17 cells transferred into mice are long-lived and self-renewing gave rise to Th1-like effector T cells, while persisting as IL-17 producing cells and controlled tumor growth [98]. This suggests that the transfer of tumor-specific Th17 cells might represent attractive antitumor therapy. It is nowadays possible to genetically modify T cells by transfecting them with the gene construct of a chimeric antigen receptor (CAR), engineered by the fusion of a single-chain variable fragment (scFv) to intracellular signalling domains of a TCR and costimulatory molecules. CAR-transfected T cells recognize a specific epitope expressed by tumor cells, without the need to be presented by MHC-I molecules. At the moment, several models of CARs have proven efficacy toward tumors both in mice [108] and in patients [109] and are evaluated in clinical trials [110]. ICOS based CARs have been shown to redirect Th17 cells to Th17/Th1 phenotype exhibiting enhanced effector functions and increased* in vivo* persistence. When transferred into tumor bearing mice (Malignant Pleural Mesothelioma (MPM)), tumor Ag specific ICOS based CAR Th17 cells induced strong tumor rejection, demonstrating that ICOS based CARS, that consequently promote Th17/Th1 plasticity, might be a promising approach in tumor immunotherapies [111].

As discussed above, the tumor microenvironment dramatically affects Th17 cell plasticity normally occurring in other inflammatory contexts; notably the conversion of Th17 cells into Treg cells is barely observed in tumors. Therefore, a better understanding of the mechanisms implicated in the maintenance of Th17 lineage of cells transferred in tumor patients would certainly improve the current protocols. In the tumors in which Th17 cells were correlated with a better outcome, an alternative strategy would be to promote plasticity from Treg cells toward a Th17 phenotype. This aim might be achieved by providing the adequate cytokinic environment (such as IL-6 and TGF-*β*), by inhibiting IDO that prevented conversion of Treg cells toward Th17 cells [62, 92] or even by combining the two strategies. Altogether, although Th17 plasticity is not yet well defined in the tumoral context, this particularity of Th17 cells might be exploited and represents interesting target for the development of future therapeutic strategies.
